# NURSING ASSISTANT TURNOVER IN HOME HEALTH CARE AGENCIES: A SCOPING LITERATURE REVIEW

**DOI:** 10.1093/geroni/igad104.3497

**Published:** 2023-12-21

**Authors:** Jennifer Wagner, Vivian Miller, Lauren Maziarz, Melissa Burek, Julia Bell, Kaley Perry

**Affiliations:** Bowling Green State University, Bowling Green, Ohio, United States; Bowling Green State University, Bowling Green, Ohio, United States; Bowling Green State University, Bowling Green, Ohio, United States; Bowling Green State University, Bowling Green, Ohio, United States; Bowling Green State University, Bowling Green, Ohio, United States; Bowling Green State University, Bowling Green, Ohio, United States

## Abstract

Certified nursing assistants (CNAs) who work in Home Health Care (HHC) are part of the continuum of care and aid in reducing the burden to the acute care system. CNAs assist patients with their activities of daily living and provide social engagement. Since CNAs see patients more frequently, they are crucial for referral to the nursing staff in times of crisis. The Affordable Care Act was enacted in 2010 which expanded HHC services and provides consumers with more health care options. There is a projected 25 percent increase in CNAs needed in HHC between 2021 and 2031. Despite the growth in HHC, the sector has high turnover rates of CNAs. It is estimated that 35 to 65 percent of CNAs leave HHC employment annually. This scoping review utilized the Job Demands-Resource (JD-R) and the Social-Ecological (SE) models to identify trends in turnover. The JD-R can be used to understand how the demands of one’s job as well as the engagement in one’s work can affect overall work-related stress, health, and motivation, and is contrasted with available job resources. The SE framework was used to explore levels of influence, and examines how individuals, relationships, communities, and society interact to influence behavior and perceptions. By using both the JD-R and SE models, it is easier to conceptualize the interaction of factors impacting turnover and that many of these factors are unique to CNAs in HHC. The difficult nature of the work, low pay, and low autonomy mean identifying job resources is critical.

